# Nonlinear MPC Design using the qLPV Approach and IQC-based Terminal Ingredients

**DOI:** 10.1007/s40313-026-01281-x

**Published:** 2026-04-30

**Authors:** Marcelo M. Morato, Tobias Holicki, Vinícius Moreno Sanches, Carsten W. Scherer

**Affiliations:** 1 Departamento de Engenharia Automação e Sistemas, Universidade Federal de Santa Catarina, Florianópolis, Brazil; 2 Univ. Grenoble Alpes, Grenoble INP, Institute of Engineering Univ. Grenoble Alpes, CNRS, GIPSA-lab, Grenoble, France; 3 Chair for Mathematical Systems Theory, Department of Mathematics, University of Stuttgart, Stuttgart, Germany

**Keywords:** Model predictive control, Lur’e systems, Linear parameter varying systems, Integral quadratic constraints, Stability

## Abstract

The design of stabilizing model predictive control laws for systems subject to nonlinearities is often handled using sector arguments. Recent advances have demonstrated that the synthesis of terminal ingredients based on integral quadratic constraints (IQCs) with general dynamic multipliers yields less conservative design—with larger corresponding regions of attraction. However, the resulting predictive control scheme involves a nonlinear prediction model and, hence, may become computationally demanding. Yet, a large body of research has shown that for (quasi-)linear parameter varying (qLPV/LPV) models, which constitute a particular class of nonlinear systems, a significant reduction of the resulting computational burden is possible. Accordingly, in this paper, we provide a systematic nonlinear MPC design procedure combining IQC-based terminal ingredient (for larger stability regions) and LPV tools (for reduced computational load). The proposed scheme is compared, in simulation, to state-of-the-art (nonlinear) MPC algorithms, indicating good control performance with lower associated numerical cost. Moreover, a real processor-in-the-loop validation experiment is provided to demonstrate the applicability of the scheme in practical engineering contexts.

## Introduction

Model predictive control (MPC) is nowadays a well -established design technique with extensions to a variety of systems and settings (Mayne, 2014; Limon et al., 2018; Andrade et al., 2024; Morato et al., 2024b). Theoretical certificates for closed-loop stability and recursive feasibility of the involved optimization problem are typically obtained by means of Lyapunov arguments (Mayne et al., 2000). The former relate to the MPC cost function and consist of the so-called *terminal ingredients*^1^ in the optimization problem (which is solved online).

In order to obtain such stability certificates for nonlinear systems, the corresponding MPC terminal ingredients are designed using standard sector arguments, cf. (Böhm et al., 2010; Tarbouriech et al., 2011; Köhler et al., 2021; Zhang and Song, 2022). In contrast, in our recent paper (Morato et al., 2023), we demonstrate how to construct such stabilizing ingredients using *integral quadratic constraints* (IQCs) with general dynamic multipliers, and illustrate that such an approach yields controllers with larger associated regions of attractions. The IQC framework is well known in the robust control literature for its capability to deal with (combinations of) various classes of uncertainties and nonlinearities, often with less conservatism than alternative approaches (Veenman et al., 2016; Scherer and Veenman, 2018; Scherer, 2023). In the aerospace industry, for instance, control applications using IQCs have consistently been exploited (Veenman et al., 2009; Palframan et al., 2017).

Despite the significant advances in terms of control performance, the nonlinear MPC (NMPC) framework from Morato et al. (2023) exhibits computational difficulties due to the involved nonlinear optimization problem. Solving such problems numerically can be prohibitive in time-critical environments. Recently, several articles have shown that for quasi-linear parameter varying (qLPV) systems^2^, specific tools can exploit the underlying structure, cf. (Coutinho et al., 2025; Oliveira et al., 2026). This permits the formulation of computationally efficient nonlinear MPC algorithms, cf. (Morato et al., 2020a; Morato, 2023) and the references cited therein. In particular, in Cisneros et al. (2016); Morato et al. (2022b) it is shown that qLPV MPC schemes can be formulated with the complexity of (sequential) quadratic programs ((S)QPs). Nowadays, these programs can be solved in real time with standard (embedded) solvers, even under strict sampling periods in the range of milliseconds.

**Contributions.** We propose a novel nonlinear MPC scheme which combines terminal ingredients based on IQCs with a qLPV prediction model. In particular, we provide a systematic design procedure that combines the offline synthesis of stabilizing feedback laws from (Morato et al., 2023) with LPV implementation tools^3^ from (Cisneros et al., 2016; Morato et al., 2022b), resulting in numerical improvements:

1. We propose a specific qLPV embedding for Lur’e systems, i.e., linear systems in feedback with a bounded nonlinearity.
2. We provide numerical comparisons of the proposed method with two NMPC schemes from the literature:
  A standard MPC scheme employing a nonlinear solver.
  A gain-scheduled LPV MPC algorithm.
  The considered methods are evaluated via a numerical simulation benchmark and a real process-in-the-loop (PIL) experiment. Our evaluations highlight the competitiveness and reduced complexity of the proposed scheme w.r.t. available formulations.

### Remark 1

Several recent works have discussed how to design MPC schemes for nonlinear systems using LPV model predictions, cf. (Cisneros et al., 2016; Abbas et al., 2021; Morato et al., 2022b, a). To our best knowledge, these works certify closed-loop stability by exploiting the standard terminal ingredient synthesis argument from (Mayne et al., 2000)—with minor modifications, e.g., parametric dependence. Herein, however, our main novelty is to provide a new perspective to this topic, showing that the recent IQC-based synthesis procedure from (Morato et al., 2023) can also be coupled to “**LPV tools**”: a parameter-dependent linear prediction model and a corresponding scheduling trajectory estimation layer. We show that this non-trivial combination ensures stability, a recursively feasible optimization procedure and fast computation of the MPC law during the implementation.

**Outline.** Section 2 recaps the relevant results on stability in MPC and the design of terminal ingredients using point-wise IQCs with storage and dynamic multipliers. Section 3 delineates the proposed modifications by including LPV tools. The realistic simulation tests related to the new algorithm, considering engineering benchmarks from the literature, are presented in Sect. 4. General conclusions are drawn in Sect. 5.

**Notation.** $\mathbb {N}$ denotes the set of positive integers, $\mathbb {N}_0:=\mathbb {N}\cup \{0\}$, and $\mathbb {N}_{[a, b]}:=\, \{i \in \mathbb {N} \, \mid \, a \le i \le b\}$. $\ell _{2e}^m$ is the space of sequences with elements in $\mathbb {R}^m$. The $(j\times j)$-identity matrix is $I_j$. The vectorization operation is denoted via $\textrm{col}(v_1, \dots , v_m) := \, (v_1^\top , \dots , v_n^\top )^\top$; the block-diagonal matrix $V_1, \dots , V_n$ on the diagonal is given by $\textrm{diag}(V_1, \dots , V_n)$. $A\, \otimes \, B$ is the Kronecker product of matrices *A* and *B*. The weighted vector norm is denoted $\Vert x \Vert _P:=\, \sqrt{x^TPx}$. The predicted value of a signal *v* at time instant $k+i$, computed based on the information available at time instant *k*, is denoted by $v(k+i\mid k)$; in particular, $v(k\mid k) = v(k)$. For a given matrix *M*, $M^{T}$ and $M^{-T}$ denote its transpose and inverse transpose matrices, respectively.

## Recap: Stabilizing MPC via IQCs

We consider well-posed Lur’e systems. Given some real matrices and an initial condition $x(0) \in \mathbb {R}^{n_x}$, these are described in state-space, for all $k \in \mathbb {N}_0$, by1Here, *x* stands for the system’s state, *u* is the control input, and *z* and *w* denote the interconnection signals w.r.t. a *known* causal nonlinearity^4^$\Delta :\,\ell _{2e}^{n_z} \rightarrow \ell _{2e}^{n_z}$. Notice that (1) represents a nonlinear dynamical system composed of a linear part interconnected with a nonlinear block.

Lur’e systems, represented as in (1), can be used to describe a wide range of practical engineering phenomena and have been consistently revisited in the literature, cf. (Sepulchre and Stan, 2005; Seron and De Doná, 2016; Aarnoudse et al., 2024). As argued by Materassi and Salapaka (2011), a large number of real (engineering) systems have this structure, such as atomic force microscopes and electromechanical systems. In Sect. 4.2, we show that the dynamics of a drone’s landing motion can also be represented in this format.

### Definition 2.1

A function $f:\, \, \mathbb {R}\rightarrow \mathbb {R}$ is said to be slope-restricted (with slope bounds $\mu \,< \,\varrho$) if $\mu \,\le \,\frac{f(y)-f(\breve{y})}{y-\breve{y}}\, \le \, \varrho$ for all $y,\breve{y} \in \mathbb {R}$. It is said to be sector-bounded if $a\,\le \,\frac{f(y)}{y}\,\le \,b$ for all $y\in \mathbb {R}$, where $a<b$ are two real sector bounds.

### Assumption 2.2

The nonlinear operator $\Delta$ in (1) is memory-less and given by2where $\phi : \mathbb {R}\rightarrow \mathbb {R}$ is a known nonlinear function that is *sector-bounded* with bounds (*a*, *b*) and *slope-restricted* with bounds ($\mu ,\varrho$), according to Definition 2.1.

Next, instead of directly analyzing the nonlinearity $\Delta$ itself, we analyze the outputs of an LTI filter $\Psi$. In particular, $\Psi$ filters the inputs *z* and outputs *w* of the nonlinear block $\Delta$ in (2), i.e., , with $\xi$ being the corresponding filter states. Accordingly, if Assumption 2.2 holds, it follows that $\Delta$ satisfies a point-wise IQC (Morato et al, 2023, Theorem 2) with appropriate multipliers and w.r.t. the stable and *a priori* chosen LTI filter $\Psi$, here given by3for all $k\in \mathbb {N}_0$ and $\xi (0)\,=\,0 \in \mathbb {R}^{n_\xi }$. To apply the IQC result from (Morato et al., 2023), we consider the augmented system4for all $k \in \mathbb {N}_0$, with $\nu : k\mapsto \textrm{col}(\xi (k), x(k))$ and the corresponding state-space matrices given by

### MPC and Stability

Given such a system setup, and with full augmented state $\nu$ measurements at any discrete-time instant $k \in \mathbb {N}$, we consider an MPC scheme that determines a stabilizing feedback law $u(k) \,=\, \kappa (\nu (k))$. The implementation of the MPC relies on solving, at each sample *k*, the following optimization problem:

5 $$\begin{aligned} &  \min _{U_k} J(\nu (k),U_k) \text {,} \nonumber \\ &  \text {s.t.: } \nu (k+j+1\mid k) = \mathcal {A}\nu (k+j\mid k) + \mathcal {B}w(k+j\mid k) \nonumber \\+ &  \mathcal {B}_u u(k+j\mid k)\,\text {,} \nonumber \\ &  z(k+j\mid k) = Cx(k+j\mid k) + D_uu(k+j\mid k)\,\text {,} \nonumber \\ &  w(k+j\mid k) = \Delta \left( z(k+j\mid k)\right) \,\text {,} \nonumber \\ &  \left( x(k+j\mid k),u(k+j-1\mid k)\right) \in \mathcal {X}\times \mathcal {U}\,\text {,} \nonumber \\ &  u(k+N_p-1\mid k) = \kappa _t x(k+N_p-1\mid k), \nonumber \\ &  \nu (k+N_p\mid k) \in \textbf{X}_t. \end{aligned}$$

In (5), the minimizer (argument) $U_k :=\, \textrm{col}\big (u(k\mid k), \dots , u(k+N_p-1\mid k)\big )$ represents the sequence of control inputs along the prediction horizon $N_p$ and the first element of the optimal solution $U_k^\star$ defines the MPC law, i.e., $\kappa (\nu (k)) = u^\star (k\mid k)$. Moreover, the cost function *J* is specified by

6 $$\begin{aligned} &  J(\nu (k),U_k):= V(\nu (k+N_p\mid k)) \nonumber \\ &  + \sum _{j=0}^{N_p-1} \ell (\nu (k+j\mid k), u(k+j\mid k)). \end{aligned}$$

It comprises a so-called stage cost $\ell$ over the prediction horizon and a terminal cost *V*, which weights the state at the end of this horizon. Herein, we consider the common quadratic cost: $\ell : \mathbb {R}^{n_\nu } \times \mathbb {R}^{n_u} \rightarrow \mathbb {R}$, $(x, u) \mapsto x^\top Q x + u^\top R u$, with positive definite matrices *Q* and *R*.

The sampled MPC optimization problem in (5) also accounts for process constraints motivated by, e.g., physical limitations of the controlled systems. These constraints are henceforth represented by the following symmetric sets $\mathcal {X}:= \,\{x \in \mathbb {R}^{n_x}:\, -\bar{x} \le x \le \bar{x}\}$ and $\mathcal {U}:=\, \{u \in \mathbb {R}^{n_u}:\, -\bar{u} \le u \le \bar{u}\}$, considering some nonnegative known bounds $\bar{x}\in \mathbb {R}^{n_x}$ and $\bar{u} \in \mathbb {R}^{n_u}$. We note that polyhedral constraints sets could also be considered without loss of generality (regarding both the optimization format and the corresponding design procedure detailed later). However, along the sequel, we keep the box-type sets, for simplicity.

The constraints in (5) involve the nonlinear operator $\Delta$, the hard state and control input constraints defined by the sets $\mathcal {X}$ and $\mathcal {U}$, a static terminal feedback law and a terminal set. The most important properties in MPC design are *recursive feasibility* of the optimization problem and *stability* of the corresponding closed-loop interconnection. Next, we adapt the criteria proposed in the seminal work by Mayne et al. (2000) to the concrete problem in (5). The arguments from Theorem 2.3 are standard and widely used in MPC literature, cf. Mayne et al. (2000); Morato et al. (2023).

In the sequel, we say that the optimization (5) is *recursively feasible* if its initial feasibility (i.e., it is feasible for the initial conditions when $k = 0$) ensures that it remains feasible for all successive samples $k \in \mathbb {N}$. Moreover, we say that the closed-loop interconnection of the Lur’e system (1) with the nonlinearity (2) and the MPC scheme (5) is stable if there exists a constant $c > 0$ s.t. $\Vert x(k)\Vert \le c, \forall k \in \mathbb {N}_0$ and $\lim _{k \rightarrow \infty } \Vert x(k)\Vert = 0$ for any initial condition $x(0)\in \mathbb {R}^{n_x}$, under the assumption that (5) is feasible at time $k = 0$ for .

#### Theorem 2.3

(Stability and recursive feasibility) Assume that the stage cost $\ell : \mathbb {R}^{n_\nu } \times \mathbb {R}^{n_u} \rightarrow \mathbb {R}$ and the terminal cost $V: \mathbb {R}^{n_\nu } \rightarrow \mathbb {R}$ are quadratic and positive definite for all $(x,u) \, \in \mathcal {X}\times \mathcal {U}$. Moreover, suppose that the following conditions are satisfied:

1. The origin lies in the interior of the terminal set $\textbf{X}_t$.
2. The terminal set $\textbf{X}_t$ is positively invariant for the system (4) in closed loop with the terminal control law $u(k)\,=\,\kappa _tx(k)$. That is, for any time instance *k*, if $\nu (k)$ is contained in $\textbf{X}_t$, then, so is $\nu (k+1)$.
3. For any closed-loop trajectory and any $k\in \mathbb {N}$ with $\nu (k) \in \textbf{X}_t$, the stage cost and terminal cost satisfy $V(\nu (k+1))-V(\nu (k)) \le - \ell (\nu (k),\kappa _t x(k))$.
4. The terminal control law is admissible, i.e., for any  we have $\kappa _t x\in \mathcal {U}$.
5. The terminal set $\textbf{X}_t$ only contains admissible states, i.e., for any  we have $x \in \mathcal {X}$.

Then, the MPC scheme (5) is recursively feasible and the resulting closed-loop interconnection is stable.

#### Sketch of Proof

Consider that there exists an invariant set $\textbf{X}_t$ related to the extended state dynamics $\nu (k)$ under the feedback law $u(k)=\kappa _t x(k)$, as stated by (C2). Furthermore, consider the cost function $J(\nu (k),U_k)$ as a candidate Lyapunov function for the corresponding dynamics. Then, $J(\nu (k+1),U_{k+1})-J(\nu (k),U_k)<0$ implies the inequality in (C3), considering that $u(k+N_p\mid k) = \kappa _t x(k+N_p\mid k)$. The remaining conditions (C1), (C4) and (C5) ensure that constraints are satisfied within the terminal region, that is,  implies that $(x,\kappa _t x)\in \mathcal {X}\times \mathcal {U}$. From optimality, recursive feasibility of the optimization (5) is established, given an initially feasible solution, i.e., there indeed exists a feasible $U_0^\star$ for the initial state $\nu (0)$. Then, closed-loop stability is also ensured and $\nu (k)$ is steered to the interior of $\textbf{X}_t$.

### IQC-Based Terminal Ingredients Synthesis

The fundamental idea of the IQC framework (Megretsky and Rantzer, 1997) for analyzing systems of the form (1) is to find numerically suitable quadratic constraints on the interconnection signals *z* and *w* enforced by $w = \Delta (z)$. Then, the linear part in (1) is analyzed w.r.t. such constraints—classically formulated in terms of so-called *multipliers*. These multipliers are given by $\Pi = \Psi ^*M \Psi$, with $\Psi$ being a fixed stable filter and a matrix $M \in \mathbb {S}^m$, which serves as a variable and is subject to suitable constraints. Many nonlinearities and corresponding multipliers can be found in the catalogs in Megretsky and Rantzer (1997); Veenman et al. (2016). Next, we recap the point-wise IQC class, as proposed by Morato et al. (2023) and the corresponding application to compute the terminal ingredients related to (5). We stress that Theorem 2.4 represents a variant of the IQC theorem presented in Scherer and Veenman (2018).

#### Theorem 2.4

(Point-wise IQCs with storage) Suppose that (1) is well posed, operating in open loop ($$u=0)$$, and that $$\Delta :\ell _{2e}^{n_z} \rightarrow \ell _{2e}^{n_w}$$ satisfies a *point-wise IQC with storage* w.r.t. some filter $$\Psi $$ and matrices $$M \in \mathbb {S}^m$$ and $$Z \in \mathbb {S}^{n_\xi }$$. That is, for all $$k\,\in \,\mathbb {N}_0$$, the inequality

7 $$\begin{aligned} \xi (k+1)^\top Z\xi (k+1) - \xi (k)^\top Z \xi (k) + y(k)^\top M y(k) \ge 0 \end{aligned}$$

holds for any state and output trajectory of the filter in (3) driven by the input $(z^\top \,\Delta (z)^\top )^\top$ and with any $z \in \ell _{2e}^{n_z}$. Then, (1) is asymptotically stable if there exists a matrix $X \in \mathbb {S}^{n_\xi +n_x}$ satisfying the following inequalities:89Moreover, the following energy decay relation holds for all $k\in \mathbb {N}_0$ and any initial condition $x(0) \in \mathbb {R}^{n_x}$:

#### Sketch of Proof

Assume that there exists an LTI filter $\Psi$ described in state-space by (3) and that the corresponding augmented system with states  is described by (4), considering a vanishing filter initial condition $\xi (0)=0\in \mathbb {R}^{n_\xi }$ and $u=0$. Then, the point-wise IQC (7) describes an exact difference dissipation inequality with the quadratic supply rate $s : \, (y,z) \mapsto y^\top My$ for the interconnection in (1). Moreover, the inequality (8) translates into a second difference dissipation inequality, this time with the quadratic supply rate $\tilde{s} : \, (y,w) \mapsto -y^\top My$. Therefore, by exploiting the particular structure of these two supply rates, both inequalities can be directly combined in order to obtain the given energy decay relation. Given the strictness of (8), asymptotic stability of interconnection in (1) is established.

Theorem 2.4 provides an IQC-based argument that ensures that the system in (1) is asymptotically stable in open loop, with $u=0$. Next, we consider the closed-loop setting with the MPC input $u(k)=\kappa (\nu (k))$ given as the first entry of the optimal solution of (5). Accordingly, based on Theorem 2.4, we can find a suitable terminal cost $V(\cdot )$ and a terminal set $\textbf{X}_t$, for a given terminal feedback gain $\kappa _t$, ensuring the properties from Theorem 2.3.

#### Theorem 2.5

(Stability and recursive feasibility via IQCs) Let $\kappa _t \in \mathbb {R}^{n_u \times n_{\nu }}$ be some static gain and suppose that the operator $\Delta$ satisfies (7) for some filter $\Psi$ and matrices $Z \in \mathbb {S}^{n_\xi }$, as well as $M\in \mathbb {S}^m$. Furthermore, consider that there exist a matrix  such that the following inequalities hold for all $j \in \mathbb {N}_{[1, n_x]}$ and $i \in \mathbb {N}_{[1, n_u]}$:1011Then, all of the assumptions in Theorem 2.3 are satisfied for:

(*i*): The terminal cost $V: \mathbb {R}^{n_\nu } \rightarrow \mathbb {R}$ given by:
(*ii*): The terminal set defined by:

Using these terminal ingredients, the MPC scheme (5) is recursively feasible and the resulting closed loop is stable.

#### Sketch of Proof

Consider that the operator $\Delta$ satisfies (7) for the closed-loop interconnection of the augmented system (4) and the terminal law $u(k) = \kappa _t x(k)$. As a direct consequence of Theorem 2.4 and the implied energy decay inequality, the first LMI in (10) assures (C2) and (C3) from Theorem 2.3, while the second LMI in (10) assures that the terminal cost  is positive definite and that $\textbf{X}_t$ contains the origin (C1). Furthermore, the LMIs in (11) ensures that state and input constraints are satisfied once the state is in the terminal set, thereby ensuring assures (C4) and (C5) from Theorem 2.3. As a result, recursive feasibility and closed-loop stability are established.

Next, by exploiting Theorem 2.5, we employ IQCs involving *dynamic* filters in order to compute the complete set of terminal ingredients. As argued in detail in Morato et al. (2023), the use of these dynamic filters within the IQC approach exhibits a strong benefit for constructing MPC terminal ingredients: The resulting scheme becomes less conservative (if compared to relying on simple static filters, e.g., classic sector arguments) and are hence associated with larger regions of attraction.

#### Proposition 2.6

(Dynamic multipliers) The inequalities (10)-(11) are satisfied for some given matrices $\mathfrak {X}$, $\kappa _t$, *Z* and *M* if and only if there exists some slack variable $F\in \mathbb {R}^{n_u \times (n_\nu +n_w+n_u)}$ satisfying12with  as well as ensuring that for all $j \in \mathbb {N}_{[1, n_x]}$ and $i \in \mathbb {N}_{[1, n_u]}$, the following inequalities hold13

Based on Proposition 2.6, we recap the IQC-based design criteria for all terminal ingredients of (5) using dynamic filters. In particular, we employ the combination of static full-block multipliers and dynamic O’Shea–Zames–Falb multipliers, which render (7) satisfied with $Z \ne 0$.

#### Corollary 2.7

(Dynamic multipliers) Let $\Psi$ be a basis filter such that $\Pi = \Psi ^*M \Psi$ is a dynamic O’Shea–Zames–Falb multiplier and suppose that $\mathfrak {X} \in \mathbb {S}^{n_\xi + n_x}$, $\kappa _t\in \mathbb {R}^{n_u \times n_x}$, $F\in \mathbb {R}^{n_u \times (n_\nu + n_w + n_u)}$ and *appropriate* matrices $(Z, M) \in \mathcal {M}_{\textrm{OZF}+\textrm{FB}}$ satisfying (13). Furthermore, assume that the MPC scheme (5) is operated using the terminal cost , the terminal set $\textbf{X}_t \,=\, \{\nu : V(\nu ) \le 1\}$ and the terminal law $\kappa _t$. Then, the MPC is recursively feasible and the closed loop is stable.

In Corollary 2.7, by *appropriate* matrices, we mean that (*Z*, *M*) belong to full-block and O’Shea–Zames–Falb sets, considering the definition originally presented in Morato et al. (2023)—as recapped below.

#### Lemma 2.8

(Static full-block multipliers, cf. Lemma 2 in Morato et al. (2023)) Let us denote by $\Delta _1, \dots , \Delta _N$ the generators of the convex set $\left\{ \textrm{diag}(\delta _1, \dots , \delta _{n_z})~\mid ~ \delta _j \in [a, b]\right. \left. \text { for all }j\right\}$. Then, the operator $\Delta$ in (2) satisfies (7) under Assumption 2.2, with sector bounds (*a*, *b*), for the static filter $\Psi = I$ with $n_\xi = 0$ and for any matrix *M* contained in the set

#### Theorem 2.9

(Dynamic O’Shea–Zames–Falb multipliers, cf. Theorem 3 in Morato et al. (2023)) Let $\psi (z):= I_{n_w} \otimes (\frac{1}{z^l}, \dots , \frac{1}{z}, 1)^\top$ for some length *l*, which admits the realization $\left[ I_{n_w} \otimes J_l, I_{n_w} \otimes e_l, I_{n_w} \otimes C_l, I_{n_w} \otimes e_{l+1} \right]$ where , ,  and where $J_l \in \mathbb {R}^{l \times l}$ is an upper Jordan block with eigenvalue zero. Then, the operator $\Delta$ in (2) satisfies (7) under Assumption 2.2, with slope bounds $(\mu ,\varrho )$, for the filter  and any pair of matricesdefined by $E\in \mathbb {R}^{ln_w \times ln_w}$ and $L\in \mathbb {R}^{(l+1)n_w \times (l+1)n_w}$ with the property that the matrix

$$\begin{aligned} \tilde{L}_{ij} \le 0 \text { ~for all~ }i \ne j \ \ \text {as well as} \ \tilde{L}\textbf{1} \ge 0 \ \ \text {and} \ \textbf{1}^\top \tilde{L} \ge 0 \text {;} \end{aligned}$$

here, $\textbf{1} \in \mathbb {R}^{(l+1)n_w}$ denotes the all-ones vector. In the sequel, we abbreviate the set of such matrix pairs by $\mathcal {M}_{\textrm{OZF}}$.

#### Sketch of Proof

Consider any discrete-time instant $k\in \mathbb {N}_0$ and output $z \in \ell _{2e}^{n_w}$, taking $w:= \Delta (z)$. Select the IQC filter with $\psi (z):= I_{n_w} \otimes (\frac{1}{z^l}, \dots , \frac{1}{z}, 1)^\top$ for some length *l*. The state $\xi$ and the output *y* of the filter  in response to  satisfy the following relation

$$\begin{aligned} \xi (k+1)^\top Z \xi (k+1) - \xi (k)^\top Z \xi (k) + y(k)^\top My(k) = y(k)^\top \Omega y(k) \, \text {.} \end{aligned}$$

By exploiting Corollary 6.10 of (Fetzer, 2017), we can determine that $y(k)^\top \Omega y(k)$ is nonnegative, which implies (7)in the point-wise IQC in Theorem 2.4. Therefore, the claims are established.

#### Lemma 2.10

(Combined static full-block and dynamic O’Shea–Zames–Falb multipliers, cf. Lemma 3 in Morato et al. (2023)) With the notation from the previous lemmas, the operator $\Delta$ in (2) satisfies (7) for the filterand any pair of matrices (*Z*, *M*) contained in

## Main Result

The theoretical results recapped in Sect. 2, borrowed from our recent work (Morato et al., 2023), focus on the computation of the terminal ingredients related to (5) using IQCs and dynamic filters. Nevertheless, a significant feature of the MPC optimization was not discussed: Due to its nonlinear prediction model, (5) takes the form of a nonlinear program, which means that it is computationally costly to solve during the closed-loop operation; the numerical cost required to determine a minimizer $U_k^\star$ of (5) grows exponentially w.r.t. $N_p$ and $(n_x+n_\xi )$. For time-critical systems operating under tight sampling periods, this means that only unsatisfactory approximations of $U_k^\star$ (and, thus, the sampled control feedback) can be found.

As a remedy, we propose some modifications that enable us to benefit from LPV MPC tools in order to convert (5) into an (S)QP which can be rapidly solved online, in real time, using (embedded) off-the-shelf solvers. The modifications that follow derive from a specific qLPV *embedding* choice, as detailed in the following theoretical result.

### Assumption 3.1

The state trajectories related to the Lur’e system in (1), given some MPC policy, are admissible, i.e., $x(k) \, \in \, \mathcal {X}$ holds for all $k \, \ge 0$.

### Lemma 3.2

(qLPV embedding for Lur’e systems) Consider a nonlinear system in the form of (1) satisfying Assumptions 2.2 and 3.1, with $D=0$. Then, the response of (1) coincides with the one of the qLPV system14with scheduling variable $\rho (k) =f_\rho (z(k))$. The embedding $f_\rho :\ell _{2e}^{n_z}\rightarrow \ell _{\infty }^{n_z}$ is defined by $f_\rho (z)(k):=\, \textrm{col}\left( \theta (z_1(k)),\dots ,\right. \left. \theta (z_{n_z}(k))\right)$, being $\theta (z_j)$ for a real number $z_j$ given by^5^:

15 $$\begin{aligned} \theta (z_j):= &  \left\{ \begin{array}{cl} \frac{\phi (z_j)}{z_j},& z_j \ne 0, \\ \lim \sup _{\xi \rightarrow 0} \frac{\phi (\xi )}{\xi }, & z_j = 0. \end{array}\right. \end{aligned}$$

The parameter-dependent state-space matrices are as follows:

16 $$\begin{aligned} \left\{ \begin{array}{rcl} A_{\scriptscriptstyle \text {qLPV}}(\rho ) & =& A + B\textrm{diag}(\rho )C \text {,} \\ B_{\scriptscriptstyle \text {qLPV}}(\rho ) & =& B_u + B\textrm{diag}(\rho )D_u \text {.} \end{array}\right. \end{aligned}$$

### Proof

We proceed by showing that the qLPV representation in (14) can be derived from (1) with $D=0$ using an appropriate scheduling rule. To this end, we use $w(k) = \Delta (z)(k) = \textrm{diag}(\rho (k))z(k)$, considering the scheduling rule in (15). Then, by closing the loop involving the signals *w* and *z*, we retrieve the qLPV embedding in (14) with the following (affine) parameter-dependent matrices:

17 $$\begin{aligned} \left\{ \begin{array}{rcl} A_{\scriptscriptstyle \text {qLPV}}(\rho ) & =& A + B\textrm{diag}(\rho )C \text {,} \\ B_{\scriptscriptstyle \text {qLPV}}(\rho ) & =& B_u + B\textrm{diag}(\rho )D_u \text {.} \end{array}\right. \end{aligned}$$

Moreover, assume that there exists a recursively feasible MPC algorithm (5) which ensures closed-loop stability and (state and input) constraints satisfaction by means of appropriate terminal ingredients (cf. Theorem 2.5). Then, under Assumption 2.2, it follows that the scheduling parameters belong to some closed and bounded set. That is, $\rho (k) =f_\rho \left( z(k)\right) \in \mathcal {P} \subset \mathbb {R}^{n_z}, \forall k\ge 0$, being $\mathcal {P}:=\,\left\{ \rho \in \mathbb {R}^{n_z} \, \mid \, \underline{\rho } \le \rho \le \overline{\rho }\right\}$ a known *scheduling set*, whose real bounds $\underline{\rho }$ and $\overline{\rho }$ can be retrieved from $\mathcal {X},\mathcal {U}$ and the sector bounds of $\Delta$. Moreover, if Assumption 3.1 also holds, improved bounds can be obtained. Finally, notice that (14) is equivalent to (1), which concludes the proof.

### Remark 2

Regarding the augmented system representation in (4) with $D=0$, notice that, if $w(k) = \Delta (z(k))$ and $z(k) = Cx(k) + D_uu(k)$, the augmented state $\nu$ in (4) coincides with the one of the qLPV system. That is, we obtain the following model:18for which the scheduling variable and embedding from Lemma 3.2 are maintained, considering the corresponding parameter-dependent matrices19

Other scheduling functions could be considered to generate qLPV embeddings the form of (14) that also represent the original Lur’e system in (1). A catalog of appropriate embeddings for different nonlinearities is available in Hoffmann and Werner (2014); moreover, differential and convex inclusions can also be used without loss of generality, cf. (Sala et al., 2019; Robles et al., 2019; Sala et al., 2021). However, the corresponding MPC become complex if, for instance, the chosen scheduling function depends on more than one system variable. The proposed qLPV embedding in (15) represents a practical and direct scheduling, particularly chosen to facilitate the application of aforementioned **LPV tools**.

Suppose that appropriate terminal ingredients are computed using dynamic multipliers and IQCs, by means of LMIs (Proposition 2.7 and Corollary 2.7)—which means that the closed-loop interconnection is stable and that the MPC optimization (5) is recursively feasible, as stated in Theorem 2.5. As already argued, given the nonlinear programming format of (5), computing the MPC law during the implementation of the scheme may be excessively demanding in numerical terms. Thus, we propose an alternative optimization problem, given in (S)QP format and accounting for the qLPV embedding from Lemma 3.2. When replacing the original nonlinear prediction constraint by (18) and dropping the scheduling function, i.e., replacing $\rho (k+j)=f_\rho (z(k+j))$ by estimates $\rho (k+j\mid k)$, we obtain the adapted optimization problem

20 $$\begin{aligned} &  \min _{U_k} J(\nu (k),U_k) \text {,} \nonumber \\ &  \text {s.t.: } \nu (k+j+1\mid k)= \mathcal {A}_{\scriptscriptstyle \text {qLPV}}(\rho (k+j\mid k))\nu (k+j\mid k) \nonumber \\ &  \phantom {=}+ \mathcal {B}_{\scriptscriptstyle \text {qLPV}}(\rho (k+j\mid k)) u(k+j\mid k)\,\text {,} \nonumber \\ &  \left( x(k+j\mid k),u(k+j-1\mid k)\right) \in \mathcal {X}\times \mathcal {U}\,\text {,} \nonumber \\ &  u(k+N_p-1\mid k) = \kappa _t x(k+N_p-1\mid k), \nonumber \\ &  \nu (k+N_p\mid k) \in \textbf{X}_t \text {.} \end{aligned}$$

Problem (20) is considerably modified w.r.t. the original one in (5):

- We extract the nonlinear model and replace it by an LPV one, which depends on estimates of the future trajectory of the scheduling variables. The direct implication of this is that the state prediction constraint no longer involves nonlinearities, that is, compared to (5), in (20) the original nonlinear constraints for $z(k+j\mid k)$ and $w(k+j\mid k)$ are no longer present.
- The modified optimization involves a much lower computational load. Indeed, since the cost function $J_k$ is quadratic, the sets $\mathcal {X}$ and $\mathcal {U}$ are affine, and scheduling parameter estimates are used, (20) takes a QP format at each sample. Solutions to these problems can be generated in real time, using (embedded) off-the-shelf solvers.

The terminal set $\textbf{X}_t$ is an ellipsoid, which means that the last constraint in (20) is quadratic. We stress that standard QP/SQP solution methods (e.g., interior point) allow for such constraints without any approximation, as discussed, e.g., in Boyd and Vandenberghe (2004).

Suppose that a scheduling trajectory estimate $\hat{P}_k:= \textrm{col}(\rho (k),\rho (k+1\mid k),\dots ,\rho (k+N_p\mid k))$ and a control sequence $U_k$ are at hand. Then, the qLPV model in (18) can be recursively applied along the future horizon window of $N_p$ samples in order to generate

21 $$\begin{aligned} \Upsilon _k:= &  \textbf{A}(\hat{P}_k)\nu (k) + \textbf{B}(\hat{P}_k)U_k \text {,} \end{aligned}$$

where $\Upsilon _k:=\, \textrm{col}\Big (\nu (k+1\mid k),\,\dots ,\,\nu (k+N_p\mid k)\Big )$ and the matrices $\textbf{A}(P_k)$ and $\textbf{B}(P_k)$ are given by

22 $$\begin{aligned} \textbf{A}(P_k):= &  \left[ \begin{array}{c} \mathcal {A}_{\scriptscriptstyle \text {qLPV}}(\rho (k)) \\ \mathcal {A}_{\scriptscriptstyle \text {qLPV}}(\rho (k+1))\mathcal {A}_{\scriptscriptstyle \text {qLPV}}(\rho (k)) \\ \vdots \\ \left( \prod _{j = 0}^{N_p-1} \mathcal {A}_{\scriptscriptstyle \text {qLPV}}(\rho (k\!+\!j))\right) \end{array}\right] \text {,} \end{aligned}$$

23 $$\begin{aligned} \textbf{B}(P_k):= &  \left[ \begin{array}{ccc}\mathcal {B}_{\scriptscriptstyle \text {qLPV}}(\rho (k)) & 0 & \dots \\ \mathcal {A}_{\scriptscriptstyle \text {qLPV}}(\rho (k))\mathcal {B}_{\scriptscriptstyle \text {qLPV}}(\rho (k)) & \mathcal {B}_{\scriptscriptstyle \text {qLPV}}(\rho (k\!+\!1)) & \dots \\ \vdots & \ddots & \vdots \\ \left( \prod _{j = 1}^{N_p-1} \mathcal {A}_{\scriptscriptstyle \text {qLPV}}(\rho (k\!+\!j))\right) \mathcal {B}_{\scriptscriptstyle \text {qLPV}}(\rho (k)) & \dots & \dots \end{array}\right] \text {.} \nonumber \\ \end{aligned}$$

That is, the qLPV prediction model in (20) can be simplified to (21) so that the future augmented state values $\nu (k+j\mid k), \, \forall j \in \mathbb {N}_{[1,N_p]}$ can be computed. Note that the matrices in (22)-(23) are computed *outside* the optimization program, at each sampling instant, based on the estimate $\hat{P}_k$. As argued in the recent theses (Cisneros, 2021; Morato, 2023), these elements can be computed readily, i.e., the associated numerical cost is reduced. Next, we recap how the scheduling trajectory estimate $\hat{P}_k$ can be generated and implemented with the NMPC.

### Scheduling Trajectory Estimates

In order to generate appropriate scheduling trajectory estimates $\hat{P}_k$ at each sampling instant during the implementation, we consider the following three approaches:

1. The gain-scheduled technique, which is very commonly used in practice, cf. (Morato et al., 2020b; Alcalá et al., 2020).
2. The iterative SQP approach, as originally proposed by Cisneros et al. (2016).
3. The recursive extrapolation method, based on local Taylor approximations^6^, as recently proposed by Morato et al. (2022b).

The two latter methods yield *convergent*^7^ estimates under relatively mild conditions, as recapped next.

**1. Gain-scheduled approach**

The so-called *frozen*/gain-scheduled approach is the most simple of the ones herein discussed. It consists of using model predictions, at each sampling instant, as if the systems was LTI, i.e., as if the scheduling parameters did not vary along the future horizon. To this end, we simply take $\hat{P}_k = \textrm{col}\left( \rho (k),\dots ,\rho (k)\right)$. We note that, despite the possibly large associated prediction uncertainty, this approach is widely used in practical applications due to its simplicity, cf. (Morato et al., 2020b; Alcalá et al., 2020).

**2. Iterative qLPV method**

The approach from (Cisneros et al., 2016) is based on solving the problem (20) multiple times per sample, while refining the scheduling trajectory estimate along the iterations. The procedure is outlined as follows:

- Each (subsample) scheduling trajectory estimate $\hat{P}_k^l$ is defined in terms of quantities from the previous iteration $l-1$ and, in particular, does not involve any variable that is subject to optimization. In particular, we stress that the outlined superscript *l* indicates the corresponding (*l*th subsample) iteration at time instant *k* of the related variable, i.e., $\hat{P}_k^0$ denotes the scheduling trajectory estimate at the fist iteration $l=0$. Hence, in each one of these iterations, a QP is solved.
- The method starts by solving (20) using the initial scheduling trajectory estimate $\hat{P}_k^0 = f_\rho (\hat{Z}_{k-1})$, considering the corresponding predicted trajectory
  $$\begin{array}{rcl} \hat{Z}_k:= &  \textrm{col}\big (z(k\mid k), z(k+1\mid k), \dots , z(k+N_p\mid k)\big )\text {.} \end{array}$$
- Then, along the consecutive iterations, the scheduling function (15) is applied over the predicted state trajectory, i.e., $P_k^l \,=\, f_\rho \left( \textrm{col}\big (\hat{Z}_{k}^{l-1}\big )\right)$.
- The iterations are typically stopped if the norm $\Vert \hat{P}_k^l - \hat{P}_k^{l-1}\Vert$ is smaller than some threshold or after a fixed number of QPs has been solved.
- In Hespe and Werner (2021), it is shown that such *iterative* strategy indeed converges. That is, the iterated estimate $\hat{P}_k^l$ converges toward the true future scheduling trajectory $P_k$ as the number of iterations *l* grows. The main drawback are the several iterations of the algorithm, which requires to solve (5) multiple times per sample. Accordingly, the control algorithm exhibits an overall complexity of an SQP. For a limited number of iterations, in general, solving the SQP can be numerically more amenable than solving a nonlinear optimization program.

**3. Recursive extrapolation qLPV method**

The scheme from (Morato et al., 2022b) is based on local Taylor expansions of the function $f_\rho (\cdot )$. Specifically, the approach admits the following features:

- The method relies on the assumption that $\phi$ is sufficiently smooth such that $f_\rho (\cdot )$ has bounded local derivatives.
- At each instant, the local first-order derivative of (15) is computed^8^ according as:
  $$\begin{aligned} f_\rho ^{\partial _z}(k):= &  \left. \frac{\partial f_\rho (z)}{\partial z}\right| _{\scriptscriptstyle z(k)} \text {.} \end{aligned}$$
- Furthermore, the following incremental trajectories are computed based on the last sampled trajectories from (20), at $k-1$, i.e., $\delta \hat{Z}_k:= \textrm{col}\left( \delta z(k+j\mid k-1)\right) ,\forall j \in \mathbb {N}_{[1,N_p-1]}$ with $\delta z(k+j\mid k-1):= z(k+j\mid k-1)-z(k+j-1\mid k-1)$.
- Then, the available data at the sampling instant *k* is used to correct biased predictions from the previous instant, i.e., $\rho (k)$ replaces $\rho (k\mid k-1)$, $\delta z(k)$ replaces $\delta z(k\mid k-1)$ and so forth.
- Finally, using the local Taylor expansion rule, the following simple *recursive* law is used to compute each future scheduling parameter estimate, $\forall j \, \in \, \mathbb {N}_{[1,N_p-1]}$:
  $$\begin{aligned} \rho (k+j\mid k) \,=\, \rho (k+j-1\mid k-1) + f_\rho ^{\partial _z}(k) \delta z(k+j-1\mid k)\,\text {.} \end{aligned}$$
- The main advantages of this recursive approach, in comparison to the iterative scheme (Cisneros et al., 2016), is that we can compute an associated (non-conservative) estimation error bound, and that it only requires the use of linear operators (thus being numerically light); moreover, the corresponding optimization algorithm has the numerical complexity of a **single** QP per sample.

In terms of the numerical complexity associated with each technique, we emphasize that the gain-scheduled approach yields an MPC scheme with an associated complexity of a single QP. No additional computation has to be done to estimate the future scheduling parameters. When using the iterative qLPV method, however, we generate an MPC scheme that requires one to solve multiple QPs per sample, i.e., it admits the associated complexity of an SQP. Finally, when the recursive method is applied, we compute, at each sample, a linear (vector) operation and then we solve a single QP.

Since $\hat{P}_k$ is typically non-exact, the predictions from (20) are biased w.r.t. true system trajectories. Appropriate robustness arguments should be included in the formulation in order to ensure recursive feasibility and stability properties. Typically, constraints tightening (tubes) and disturbance propagation arguments are used, cf. (Hanema et al., 2021; Morato et al., 2022c, 2024a). Several works have shown that the resulting uncertainty using the recursive and iterative LPV tools are *small* and vanish over time (Hespe and Werner, 2021; Morato et al., 2024a). For simplicity, we disregard the uncertainty in relation to $\hat{P}_k$ in the proposed MPC (20). In the simulation examples provided in Sect. 4, we show that the uncertainty is indeed negligible.

### The Complete Algorithm

In order to conclude, we summarize the steps required to apply the proposed MPC scheme:

**Offline preparations**:

1. Given a Lur’e system described by the linear model in (1) and the known nonlinear operator $\Delta$ in (2), choose an LTI filter $\Psi$ as in (3) based on a dynamic O’Shea–Zames–Falb multiplier (Corollary 2.7).
2. Then, given the augmented system description in (4), choose positive definite MPC tuning weights (matrices *Q* and *R*).
3. Given the MPC weights, the system constraints sets ($\mathcal {X}$ and $\mathcal {U}$), and the dynamic O’Shea–Zames–Falb multiplier, compute the stabilizing terminal ingredients $\big (\kappa _t(\cdot ),V(\cdot ),\textbf{X}_t\big )$ that satisfy Theorem 2.5 by solving the corresponding LMIs in Proposition 2.7 and Corollary 2.7.
4. Using Lemma 3.2 and Remark 2, generate the qLPV models for the system and the augmented dynamics, and set up the MPC optimization problem in the format of (20).

**Online implementation** (for each sample discrete time *k*):

1. Measure the system state *x*(*k*) and the interconnection signals *z*(*k*) and *w*(*k*),
2. Compute the stacked state $\nu (k)$ and the sampled scheduling variable $\rho (k)$ by applying (15).
3. Generate a scheduling trajectory estimate $P_k$ (using, either the gain-scheduled, the iterative, or the recursive qLPV approach).
4. Solve (20) and apply the first entry of a minimizer $U_k^\star$ as the local MPC feedback $\kappa (\nu (k))$.

## Realistic Simulation Benchmarks

In this section, we seek to demonstrate the effectiveness of the proposed MPC solution using IQCs and LPV tools by means of numerical simulations. In particular, we consider two different nonlinear benchmark examples from the literature to show that our approach enables good control performance with reduced computational cost. First, we present results considering the regulation problem of a nonlinear example with a sinusoidal feedback (Sect. 4.1). Then, a real processor-in-the-loop validation experiment is presented, considering a kinematic drone landing problem (Sect. 4.2).

The numerical results presented in the sequel were achieved using MATLAB software and Yalmip toolbox on a M1 Pro 16 GB RAM Macintosh computer. All LMIs were solved (offline) using SDPT3 (Tütüncü et al., 2001).

### Sinusoidal Nonlinear Example

We consider the regulation problem for a discrete-time Lur’e system, considering a sampling period of $T_s = 100$ ms, with a sinusoidal feedback nonlinearity $w(k):=\,\sin (z(k))$ and the state-space matrices $D=0$ and24To this end, we employ four distinct MPC schemes:

- A nonlinear MPC using a min–max approach, as in, e.g., Limón et al. (2006). The method is implemented using the built-in branch-and-bound solver from MATLAB, namely BNB, and denoted by “min–max NMPC.”
- A standard gain-scheduling LPV MPC scheme, using parameter-dependent terminal ingredients (Morato et al., 2021) and gurobi as solver, henceforth denoted by “Standard LPV MPC.”
- Two versions of the proposed scheme, using dynamic IQCs and LPV tools, also using gurobi as solver:
  - The iterative qLPV approach, with an iteration limit of $n_{\text {iter}}=2$, henceforth denoted by “Iterative IQC LPV MPC.”
  - The recursive extrapolation approach, henceforth denoted by “Recursive IQC LPV MPC.”

In terms of the latter three schemes, we employ Lemma 3.2 to obtain the qLPV model in (14), in accordance with the scheduling function in (15), which yields:

25 $$\begin{aligned} \rho _j(k):= &  \frac{\sin (z_j(k))}{z_j(k)}, \forall j \in \mathbb {N}_{[1,3]}\text {.} \end{aligned}$$

In terms of process constraints, we use $\mathcal {X}:=\, \left\{ x \in \mathbb {R}^3\, \mid \,\right. \left. \vert x_j\vert \,\le \, 2, \forall j \in \mathbb {N}_{[1,3]}\right\}$ and demand the control to belong to $\mathcal {U}:=\, \left\{ u \in \mathbb {R}\, \mid \, \vert u \vert \le 3\right\}$. The four MPC algorithms were tuned using $Q \,=\,\textrm{diag}\left( 0.5,0.5,0.6\right)$, $R\,=\,0.8$ and a prediction horizon of $N_p\,=\,7$ steps.

**Fig. 1:**
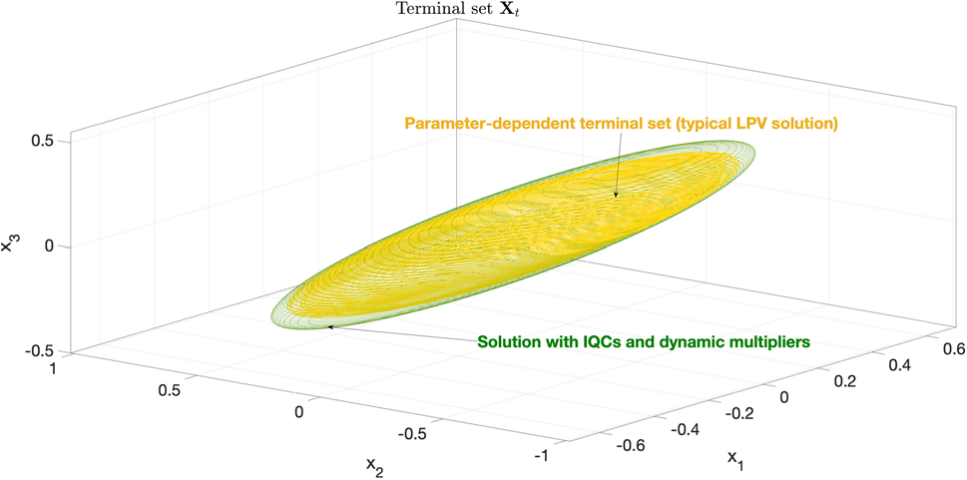
Terminal set $$\textbf{X}_t$$

First, we evaluate the stability ingredients using standard parameter-dependent (LPV) sets and the novel ones obtained using IQCs and dynamic multipliers (Theorem 2.5). The corresponding terminal sets $\textbf{X}_t$ are respectively presented in Fig. 1. Evidently, as already indicated in Morato et al. (2023), the use of the point-wise IQCs with dynamic multipliers offers a larger set, thus associating the corresponding MPC algorithm with a larger region of attraction. In particular, the trace of the corresponding positive definite matrix is $22.66\%$ smaller with the IQC solution, which implies in a $29\,\%$ larger set (Table 1).

**Table 1:** Volume of the terminal set $\textbf{X}_t$

Method	(Average) trace
LPV Terminal Ingredients	42.68
Dynamic Multipliers and IQCs	$\mathbf {33.42}$

Bold value indicates the smaller associated average computational time

**Fig. 2:**
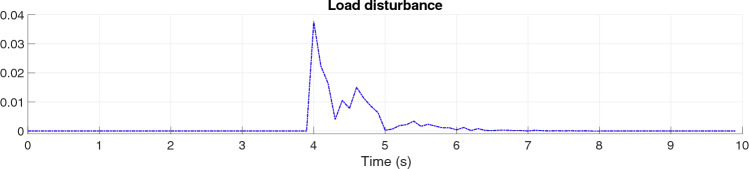
Load disturbance signal

**Fig. 3:**
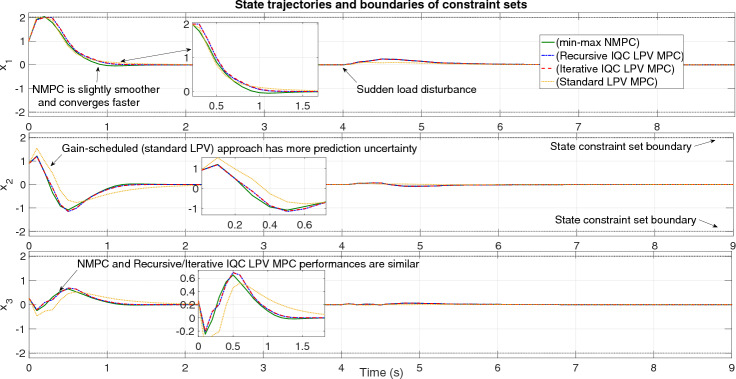
Closed-loop state trajectories

**Fig. 4:**
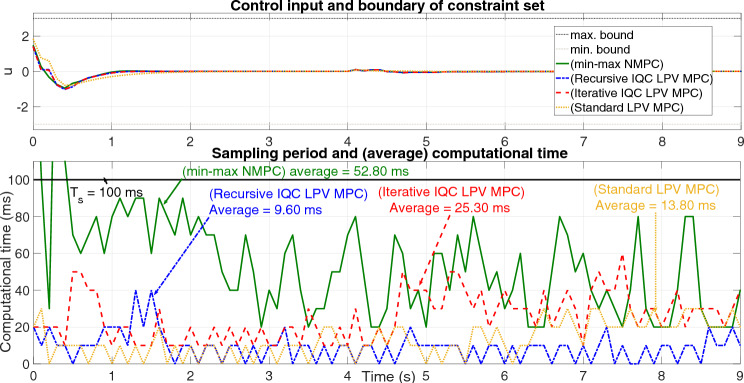
Control signal

Given the wider terminal set generated by using the dynamic multipliers and the IQC-based approach (Fig. 1), it is implied that the resulting MPC algorithm is able to stabilize a larger number of initial conditions. Note that the terminal set is linked to the region of attraction of the control scheme, as discussed in Morato et al. (2023). Moreover, by using the terminal set $\textbf{X}_f$ computed via IQCs in the MPC optimization, it possibly yields a less conservative control.

Next, we evaluate the regulation response of the closed-loop system departing from the initial condition $x(0)=\textrm{col}\big (1,0.9,0.26\big )$. Moreover, we consider that a nonpersistent load disturbance of 0.037 (maximal) absolute value is suddenly added to each of the state dynamics at $t=4$ s, as shown in Fig. 2.

**Fig. 5:**
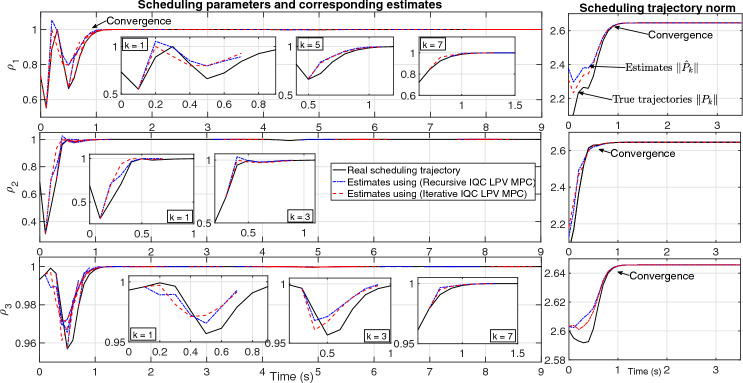
Scheduling trajectories estimates $\hat{P}_k$ using iterative (red) and recursive (blue) LPV approaches

The obtained state trajectories, considering each of the considered control approaches, are presented in Fig. 3. The corresponding control signals and the respective average computational time required to solve the MPC optimization (online) are exhibited in Fig. 4. These results are discussed next:

- As expected, the four MPC schemes are able to admissibly regulate the state trajectories (respecting inputs and state bounds), with a recursively feasible optimization.
- In terms of performance, the control results are arguably similar, with the NMPC solution exhibiting smoother^9^ and faster regulation and the gain-scheduled/frozen LPV approach the slowest—which is expected, given the large uncertainty^10^ that arises using the frozen scheduling estimates.
- In numerical terms, Table 3 presents the root-mean-square (RMS) index related to each state and to the optimal MPC stage cost $\ell ^\star _k:= \ell (x(k),u^\star (k\mid k))$, as well as the TV index, which measures the total variance of the control signal over time (i.e., $\sum _{k=0}^T\Vert u(k+1)-u(k)\Vert$). These indices are use to quantitatively assess the obtained performance.
- Both IQC LPV MPC approaches achieve comparable performance (using the iterative mechanism, Sect. 3.1.2, and the recursive scheduling estimate, Sect. 3.1.3) that almost coincide with those from the full nonlinear solution.
- The corresponding scheduling trajectory estimates $\hat{P}_k$ and the true scheduling signal as well as their norms are shown are shown in Fig. 5. This figure illustrates that the estimates indeed converge toward the true scheduling values. Clearly, convergence is rapidly established and the resulting uncertainty with both methods is similar, i.e., the residual between the true scheduling signal and the estimated one over the prediction horizon window is indeed very small. We also emphasize that the residual grows larger during two transient moments, both at the initial samples and, later on, at the load disturbance moment. We emphasize that the real scheduling signal $\rho (k)$ is computed directly from the system variables using (15) in Lemma 3.2. That is, given the current output value *z*(*k*), a linear combination of the state measurement and control input, we compute the sampled scheduling parameters using $\rho (k)=f_\rho (z(k))$.
- Another feature to analyze is the computational time $t_c(k)$ required to determine the sampled control law *u*(*k*) with each MPC algorithm. In this regard, we highlight that the min–max NMPC scheme is the most computationally expensive approach. The iterative IQC LPV MPC scheme is also relatively costly, since it requires solving $n_{\text {iter}}$ QPs per sample. Both the standard LPV MPC and the recursive IQC LPV MPC algorithm represent the “cheapest” solutions, exhibiting average computational times with the same order of magnitude. We note that both these algorithms only require to solve one QP per sample. The recursive approach also requires to solve linear equations to compute $\hat{P}_k$ ( with residual associated computational effort). In practice, the latter is able to maintain a low numerical cost while the resulting performance is very close to the one of the min–max NMPC scheme (Table 2).

**Table 2:** Average computational time required to generate each predictive control law

Method	$t_c$
(min–max NMPC)	52.80 ms
(Standard LPV MPC)	13.80 ms
(Iterative IQC LPV MPC)	25.30 ms
(Recursive IQC LPV MPC)	$\mathbf {9.60}$ **ms**

Bold value indicates the smaller associated average computational time

**Table 3:** Control performance

Method	$\ell _k^\star$	$x_1$	$x_2$	$x_3$	TV
(min–max NMPC)	$\mathbf {1.710}$	0.3939	0.2544	0.1247	$\mathbf {3.895}$
(Standard LPV MPC)	2.885	0.3911	0.2668	0.1301	3.6752
(Iterative IQC LPV MPC)	$\mathbf {2.192}$	0.4120	0.2568	0.1313	$\mathbf {3.880}$
(Recursive IQC LPV MPC)	$\mathbf {2.190}$	0.4107	0.2584	0.1313	$\mathbf {3.9248}$

Bold values indicate the smaller performance cost and TV

### Drone Landing Problem

In order to illustrate the applicability of the proposed method in a practical engineering context, we consider a realistic processor-in-the-loop (PIL) validation experiment of a drone landing problem. To this end, our LPV MPC scheme is embedded into an ESP32 microcontroller.

The PIL experiment is conducted as follows: We simulate the dynamics of an autonomous drone performing a landing maneuver in MATLAB. In parallel, our LPV MPC algorithm using IQCs runs on an ESP32 microcontroller (Cameron, 2023) implemented in C/C++ syntax. At each sampling instant, the “state measurements” (generated from the simulation model) are sent to the ESP32 board using a SERIAL port. Then a corresponding predictive control input is computed on the board and sent back to MATLAB.

The ESP32 is a low-cost Arduino-UI-compatible board with limited memory and processing capacity. Specifically,^11^ it operates under a Tensilica Xtensa 32-bit LX6 microprocessor with 520 KB SRAM and 4 MB flash memories. The implementation of the LPV MPC optimization is handled using the logarithmic-barrier interior-point solver proposed^12^ in Sanches et al. (2025).

**Fig. 6:**
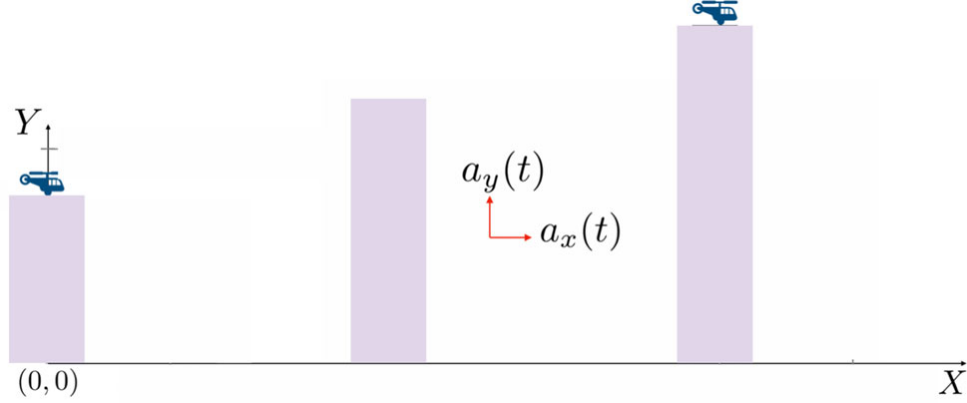
Simplified drone landing problem

The drone landing problem, illustrated in Fig. 6, is described as follows: A drone detaches from the surface of a building (i.e., at $X = 0\,\text {m}$, $Y = 50\,\text {m}$) and, from that point on, it is accelerated along the *X* and *Y* axes by an onboard autonomous acceleration system. The purpose of the onboard system (our LPV MPC scheme embedded to the ESP32 microcontroller) is to ensure that the drone lands properly a hundred meters to the right of the departure base, specifically, at $X = 150\,\text {m}$, $Y = 150\,\text {m}$.

This unmanned aerial vehicle has an inertial unit that measures the drone’s velocity and acceleration—as well as their orthogonal decompositions. The onboard system in use regulates the voltage that controls the drone’s propellers. For simplicity, it is assumed that the system directly determines the signals $a_x(t)$ and $a_y(t)$—the desired accelerations w.r.t. the *X* and *Y* axes. The kinematic behavior of the drone, in terms of velocities ($v_x$, $v_y$) and positions ($p_x$, $p_y$) w.r.t. the *X* and *Y* axes, is represented, in discrete time, by the following Lur’e model

26 $$\begin{aligned} \overbrace{\begin{bmatrix} p_x(k + 1) \\ v_x(k+1) \\ p_y(k+1) \\ v_y(k+1) \end{bmatrix}}^{x(k+1)}= &  \overbrace{\begin{bmatrix} 1 & T_s & 0 & 0 \\ 0 & 1 - k_x & 0 & 0 \\ 0 & 0 & 1 & T_s \\ 0 & 0 & 0 & 1 -T_sk_y \end{bmatrix}}^{A}x(k) + \overbrace{\begin{bmatrix} 0 & 0 \\ -T_sk_x & 0 \\ 0 & 0 \\ 0 & -T_sk_y \end{bmatrix}}^{B}w(k) \nonumber \\+ &  \underbrace{\begin{bmatrix} 0 & 0 \\ T_s & 0 \\ 0 & 0 \\ 0 & T_s \end{bmatrix}}_{B_u} \underbrace{\begin{bmatrix} a_x(k) \\ a_y(k) \end{bmatrix}}_{u(k)} + \underbrace{\begin{bmatrix} 0 \\ 0 \\ 0 \\ -T_sg \end{bmatrix}}_{w_g} \text {,} \nonumber \\ z(k)= &  \underbrace{\begin{bmatrix} 0 & 1 & 0 & 0 \\ 0 & 0 & 0 & 1 \end{bmatrix}}_{C}x(k) \end{aligned}$$

where *g* denotes the gravitational constant and $k_x=0.05$ and $k_y=0.02$ are dimensionless friction coefficients. The system states are the positions and velocities along both axes, while the nonlinear feedback is given by:

27 $$\begin{aligned} w(k)= &  \Delta (z(k)) \,=\, \textrm{col}\left( \begin{array}{cc}z_1(k)(z_1(k)-1),&z_2(k)(z_2(k)-1)\end{array}\right) \text {.} \nonumber \\ \end{aligned}$$

The nonlinear term in (27) originates from the air resistance against the drone’s motion w.r.t. both axes.

This kinematic drone landing process accounts for the following operational constrains:

$$\begin{aligned} \mathcal {X}= &  \left\{ x \in \mathbb {R}^4:\begin{array}{rcl}x_1 & \!\in \!& [-10,170]\text {m},\\ x_2 & \!\in \!& [-50,50]\text {m/s},\\ x_3 & \!\in \!& [0,170]\text {m},\\ x_4 & \!\in \!& [-15,15]\text {m/s}\end{array} \right\} , \\ \mathcal {U}= &  \left\{ u \!\in \! \mathbb {R}^2: \begin{array}{rcl}\vert u_1\vert & \le & 50\text {m/s}^2, \\ \, \vert u_2\vert & \le & 50\text {m/s}^2 \end{array}\right\} . \end{aligned}$$

In order to account for the gravitational term, the $+w_g$ bias is summed to the model prediction in the proposed optimization (20). Moreover, since the landing problem requires the MPC to establish $\lim _{k\!\rightarrow \!+\infty }p_x(k)\!=\! 150\,\text {m}$ and $\lim _{k\!\rightarrow \!+\infty }p_y(k)= 150\,\text {m}$, we make standard modifications: the considered cost function and terminal constraints in (6) are altered to account for the state deviation from the target, i.e., both the terminal cost $V(\nu )$ and the stage cost $\ell (\nu ,u)$ account for $(\nu - \nu _r)$ instead of $\nu$, where $\nu _r = \textrm{col}(0,x_r)$ and $x_r = \textrm{col}(p_{x_r},0,p_{y_r},0)$ is the steady-state target related to the target $(X,Y) = (p_{x_r},p_{y_r})$, the terminal control law is imposed as $u(k+N_p-1 \mid k) = \kappa _t (x(k+N_p-1 \mid k)-x_r)$ and the terminal constraint as $(\nu (k+N_p-1 \mid k)-\nu _r) \in \textbf{X}_t$. Also, due to the polyhedral format of the constraints set, the synthesis LMIs were accordingly altered.

In our experimental results, as discussed next, we use $p_{x_r}= 150$ m as a constant horizontal target and the vertical target $p_{y_r}$ as a step function, changing from 100 m to 160 m when the horizontal position is near its target (when $x_1(t)>140$ m) and then changing from 160 m to 150 m when the horizontal target is reached, for a smooth landing maneuver.

#### Implementation Steps

In order to apply our NMPC scheme, it is required to compute the IQC-based terminal ingredients. Accordingly, the first (offline design) step is to synthesize a feedback gain $\kappa _t$ such that the closed-loop dynamics given by $x(k+1) = (A+B_u\kappa _t)x(k) + Bw(k)$ are asymptotically stable. We emphasize that traditional LTI synthesis tools can be used here such as LQR.

To this end, considering the Lur’e model (26), under the sampling period of $T_s = 100$ ms, we tune our MPC using a prediction horizon of $N_p = 10$ samples and the following performance weights:

$$\begin{aligned} Q= &  \textrm{diag}(100, 1, 10, 0.1)\,\text {,} \\ R= &  \textrm{diag}(5,5)\,\text {.} \end{aligned}$$

**Fig. 7:**
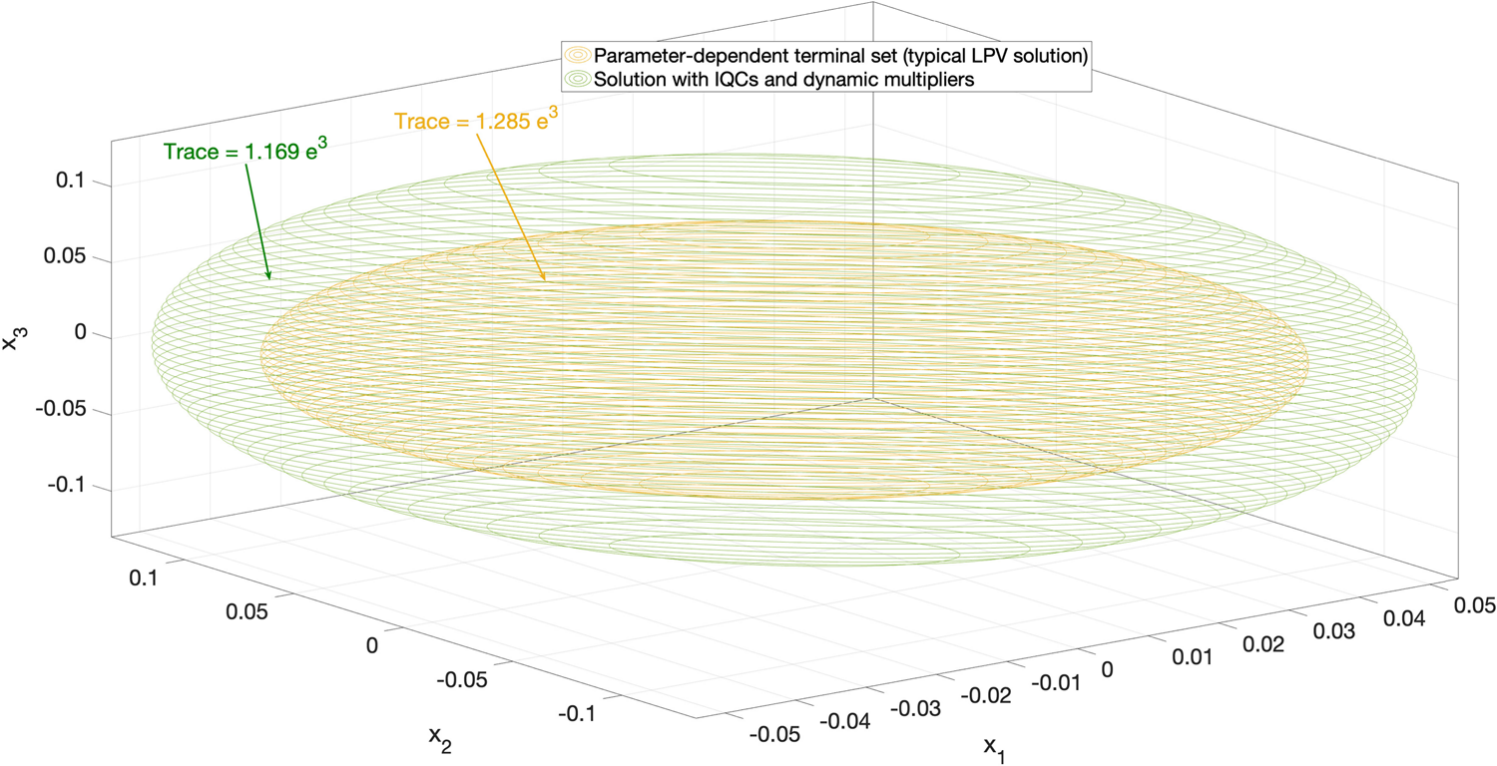
Terminal set $\textbf{X}_t$—drone landing problem

With the stabilizing gain $\kappa _t$ at hand, the second step resides in computing a terminal cost $V(\cdot )$ and a terminal invariant set $\textbf{X}_{t}$ satisfying the inequalities in Theorem 2.5. The (worst-case) involved sector bounds related to the nonlinearity in (27) are $a = -51$ and $b = 49$. These were obtained by evaluating (27) w.r.t. the system’s operational constraints ($\mathcal {X}$ and $\mathcal {U}$ sets) and the corresponding variation bounds for the state dynamics due to (26) (Fig. 7).

The third (and final) offline implementation step is to apply the proposed qLPV embedding (Lemma 3.2). In particular, since $\left. \left( \frac{ z(z-1)}{z}\right) \right| _{z=0}$ is bounded and well defined for all $x\in \mathcal {X}$, given that $z = Cx$, we consider the following direct scheduling function according to Lemma 3.2:

28 $$\begin{aligned} \rho (k) \,=\, f_\rho (z)(k)= &  \begin{bmatrix} (z_1(k)-1)\\ (z_2(k)-1) \end{bmatrix} \, \in \, \overbrace{[-1,\,49] \times [-1,\,14]}^{\mathcal {P}} \text {.} \nonumber \\ \end{aligned}$$

This scheduling parameter yields the qLPV embedding with $A_{\scriptscriptstyle \text {qLPV}}(\rho (k)) = A+B\rho C$ and $B_{\scriptscriptstyle \text {qLPV}}(\rho (k)) = B_u+B\rho D_u = B_u$.

Notice that, with the IQC-based terminal ingredients ($\kappa _t$, $V(\cdot )$, and $\textbf{X}_t$) and the qLPV embedding model, we can directly express the NMPC optimization in the proposed quadratic format of (20). Then, during the embedded (online) implementation of the predictive control scheme, the following actions have to be performed by the ESP32 microcontroller at each sample *k*:

- Compute the scheduling trajectory estimate $\hat{P}_k$—using the gain-scheduled, iterative, or recursive approach, as detailed in Sect. 3.1.
- Solve (20) and apply the first entry of the solution $U_k^\star$ as the sampled control law.

We stress that the major difficulties involved in setting up the proposed NMPC scheme are, given the sector and slope bounds ($a,b,\mu ,\varrho$), to determine the IQC-based terminal ingredients (which involves solving some LMIs) and to obtain the qLPV embedding by means of Lemma 3.2. (In this case study, the corresponding scheduling parameters arise very directly.)

With relation to the scheduling estimation mechanism, in this example, we compute the scheduling trajectory $\hat{P}_k$ using the iterative method from Cisneros et al. (2016). Due to the reduced numerical capacity of the ESP32 microcontroller, we limit the method to only two iterations of the QP per sample—thus simplifying it and decreasing the involved computational time. For this process, this iteration threshold is sufficient to ensure convergence of the scheduling parameter estimates with negligible uncertainty.

#### Control Performance

**Fig. 8:**
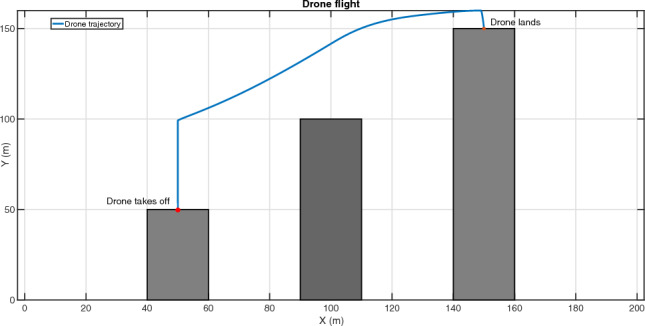
Drone’s motion along (*X*, *Y*)-plane

**Fig. 9:**
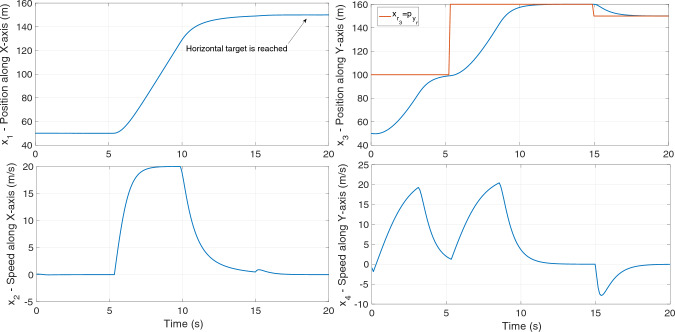
Drone landing problem: Closed-loop state trajectories

In terms of the obtained control performance with the proposed LPV NMPC scheme in the PIL experimental setting, we first show, in Figure 8, the resulting drone trajectory in the (*X*, *Y*)-plane, which indicates a smooth landing. In Figure 9, we present the state trajectories and the corresponding vertical steady-state target $p_{y_r}$ over time . Indeed, we can observe that the proposed scheme is able to ensure that these reference targets are reached and (state) constraints are respected.

**Fig. 10:**
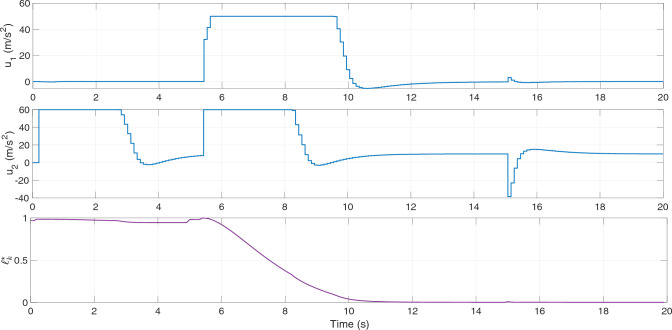
Drone landing problem: Control signal and optimal cost

Furthermore, Figure 10 presents the corresponding (desired horizontal and vertical accelerations) control inputs, which also abide to constraints. This figure also shows the normalized optimal stage cost $\ell ^\star _k$ over time, illustrating the dissipative energy decay characteristic of this (Lyapunov) function, as expected from the IQC-based terminal ingredients.

Table 4 presents the following metrics w.r.t. the observed dynamics: The normalized RMS indices related to the optimal cost and to each state trajectory, and the total variance of each control signal. The observed values indicate coherence with the selected performance weights in the MPC cost function. We recall that the last entry of *Q* is the smallest one, which means the dynamics related to the fourth state are “less important” to be minimized within the optimal criterion $J_k$.

**Table 4:** Drone landing PIL experiment: Control performance

$\ell ^\star _k$	$x_1-p_{x_r}$	$x_2$	$x_3-p_{y_r}$	$x_4$	TV($u_1$)	TV($u_2$)
0.5755	0.4126	0.4518	0.1644	0.4384	2.3924	6.1287

**Fig. 11:**
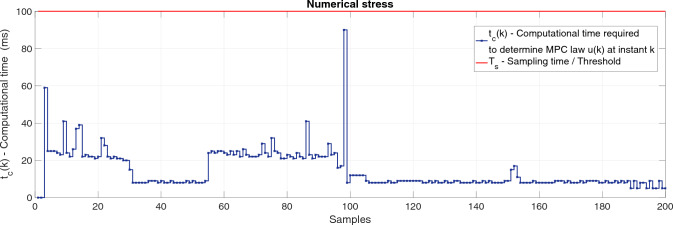
Drone landing problem: Involved numerical complexity on the ESP32 board

#### Numerical Aspects and Overall Discussion

As shown in the previous subsection, the MPC is able to track the drone landing target, i.e., stabilize the tracking error dynamics, thanks to the IQC-based terminal ingredients. Moreover, considering numerical aspects, the proposed NMPC also performs well: Solving the involved optimization problem takes, in average, 14.51 ms on the low-cost ESP32 board. In particular, Figure 11 presents the observed values for the computational time $t_c(k)$ to determine each optimal predictive control law $u^\star (k)$ during the implementation and the sampling period threshold of 100 ms.

Finally, we emphasize that our PIL experimental results were produced with a commercial low-cost microcontroller and a standard (logarithmic-barrier interior-point) optimization algorithm. This indicates that our NMPC scheme is capable to operate in real engineering context, running in real-time environments under tight sampling periods. In particular, the proposed optimization problem is solved and the control law is determined every $T_s = 100$ ms. The proposed approach to handle the nonlinear prediction by means of a specific qLPV embedding (Lemma 3.2) serves to drastically reduce the involved numerical complexity, making what was originally a nonlinear optimization problem, as in Morato et al. (2023), take the form of a few QPs, easily handled by off-the-shelf solvers.

## Conclusions

In this work, we proposed modifications to the stabilizing nonlinear MPC framework proposed in Morato et al. (2023) in order to accelerate its online implementation. In particular, we kept the terminal ingredients using IQCs with storage and proposed a prediction model modification using appropriate qLPV embeddings together with corresponding scheduling trajectory estimation tools. As a result, we offer a systematic algorithm to compute stabilizing ingredients using LMIs (offline part) with an online implementation that requires the solution of one/some QPs (rather than a nonlinear or a min–max program).

In both the numerical simulation and experimental processor-in-the-loop real-time settings, we demonstrate that the framework indeed speeds up the computation of the control action, while ensuring a similar performance. Our key finding is that the proposed method offers a computationally cheap stabilizing MPC approach that allows one to account for nonlinear dynamics. The proposed method can be of considerable interest for time-critical, embedded applications of nonlinear and time-varying processes.

## Data Availability

No datasets were generated or analyzed during the current study.

## Footnotes

1 Including a quadratic cost associated with the state at the end of the prediction horizon (terminal cost), a corresponding constraint (terminal set) and a (terminal) feedback law to the optimization problem.

2 In the LPV literature, the prefix “*quasi*” indicates that the nonlinearity is related to *endogenous* system variables (Hoffmann and Werner, 2014; Sename et al., 2013).

3 Two distinct LPV MPC techniques are taken into account: (*i*) the iterative (SQP) approach by Cisneros et al. (2016), which relies in solving the MPC optimization multiple times per sample, recursively refining the future scheduling trajectory estimates by applying the qLPV function over the optimized predictions; and (*ii*) the method by Morato et al. (2022b), based on local Taylor expansions of the scheduling function and extrapolation along the prediction horizon.

4 $\Delta$ is considered square (i.e., it has the same number of input and output channels) due to the used IQCs. This condition is a restriction of the proposed method.

5 The definition of $f_\rho (z)$ at $z=0$ is only relevant for smoothness assumptions on $f_\rho (\cdot )$ as addressed later.

6 The method in Morato et al. (2022b) is based on local approximations of the scheduling function $f_\rho (\cdot )$. An alternative approach, which to our best knowledge has not been tested, would be to apply a Taylor argument directly on the nonlinear feedback $\Delta (\cdot )$ and then estimate its future values along the prediction horizon. We leave this possibility as an open topic for future work.

7 By this, we mean that the scheduling trajectory estimates $\hat{P}_k$ converge toward the true scheduling behavior $P_k$, i.e., $\lim _{k\rightarrow +\infty } \Vert \hat{P}_k - P_k\Vert \rightarrow 0$.

8 It is implied that, from the MPC viewpoint at each sample, the variation rate of the scheduling parameter is roughly uniform, i.e., the validity of this claim is thoroughly discussed in Morato et al. (2022b). Given some sufficient conditions, it is debated therein how the scheduling estimates converge despite the mismatches between the local sampled derivative $f_\rho ^{\partial _z}$ and the true ones that vary along the prediction horizon.

9 By “smoother” we mean that the state trajectories exhibit a response with a smaller settling time and more damping.

10 The larger uncertainty with the gain-scheduled approach impacts the regulation settling time, which can be particularly observed in the trajectory of the second state in Fig. 3.

11 Full technical specs can be found in the dedicated documentation repository, cf. https://docs.espressif.com/projects/esp-dev-kits/en/latest/esp32/esp32-devkitc/index.html

12 The experimental PIL results presented herein are different from those in Sanches et al. (2025): Different process constraints are considered, a different landing trajectory is used, and the proposed IQC-based terminal ingredients are included. The used optimization solution method is the same: an interior-point logarithmic-barrier approach.
